# Angiostatin anti-angiogenesis requires IL-12: The innate immune system as a key target

**DOI:** 10.1186/1479-5876-7-5

**Published:** 2009-01-14

**Authors:** Adriana Albini, Claudio Brigati, Agostina Ventura, Girieca Lorusso, Marta Pinter, Monica Morini, Alessandra Mancino, Antonio Sica, Douglas M Noonan

**Affiliations:** 1 Polo Scientifico e Tecnologico, IRCCS Multimedica, Milan, Italy; 2 Istituto Nazionale per la Ricerca sul Cancro, Genova, Italy; 3 Laboratorio di Biologia Vascolare, CBA-Centro Biotecnologie Avanzate, Genova, Italy; 4 Dipartimento di Scienze Cliniche e Biologiche, Università degli Studi dell'Insubria, Varese, Italy; 5 Laboratorio di Immunologia Molecolare, Istituto Clinico Humanitas, Milan, Italy; 6 DISCAFF, University of Piemonte Orientale A. Avogadro, Novara, Italy

## Abstract

**Background:**

Angiostatin, an endogenous angiogenesis inhibitor, is a fragment of plasminogen. Its anti-angiogenic activity was discovered with functional assays in vivo, however, its direct action on endothelial cells is moderate and identification of definitive mechanisms of action has been elusive to date. We had previously demonstrated that innate immune cells are key targets of angiostatin, however the pathway involved in this immune-related angiogenesis inhibition was not known. Here we present evidence that IL-12, a principal TH1 cytokine with potent anti-angiogenic activity, is the mediator of angiostatin's activity.

**Methods:**

Function blocking antibodies and gene-targeted animals were employed or in vivo studies using the subcutaneous matrigel model of angiogenesis. Quantitative real-time PCR were used to assess modulation of cytokine production in vitro.

**Results:**

Angiostatin inhibts angiogenesis induced by VEGF-TNFα or supernatants of Kaposi's Sarcoma cells (a highly angiogenic and inflammation-associated tumor). We found that function-blocking antibodies to IL-12 reverted angiostatin induced angiogenesis inhibition. The use of KO animal models revealed that angiostatin is unable to exert angiogenesis inhibition in mice with gene-targeted deletions of either the IL-12 specific receptor subunit IL-12Rβ2 or the IL-12 p40 subunit. Angiostatin induces IL-12 mRNA synthesis by human macrophages in vitro, suggesting that these innate immunity cells produce IL-12 upon angiostatin stimulation and could be a major cellular mediator.

**Conclusion:**

Our data demonstrate that an endogenous angiogenesis inhibitor such as angiostatin act on innate immune cells as key targets in inflammatory angiogenesis. Angiostatin proves to be anti-angiogenic as an immune modulator rather than a direct anti-vascular agent. This article is dedicated to the memory of Prof Judah Folkman for his leadership and for encouragement of these studies.

## Background

Angiostatin is a large peptide fragment of plasminogen endowed with anti-angiogenic properties originally isolated from the urine of tumor-bearing mice [1,2]. Angiostatin and related forms consisting of the first 1–5 kingles in plasminogen (here termed collectively AST) is generated by the action of diverse proteases, including metalloproteases (MMP2, MMP12, MMP9) and serine proteases (PSA, neutrophil elastase) [3,4]. These enzymes are subject to precise regulation, and are typically activated during tumor invasion, angiogenesis and inflammation, thus AST is produced only under certain conditions and it could represent an important modulator of homeostatic responses. In vivo, AST inhibits tumor growth and keeps experimental metastasis in a dormant state [5]. AST concentrations are elevated in fluids of animals harboring primary tumors [6] and other inflammatory and degenerative diseases [7,8].

Following identification with in vivo studies, numerous in vitro studies have sought to identify the effects of AST on endothelial cells. AST has been demonstrated to produce an array of events ranging from apoptosis/activation of endothelium to inhibition of endothelial cell migration, [9-12] and tube formation [13]. Potential endothelial cell surface angiostatin receptors identified to date include cell surface ATP synthase, angiomotin and various integrins (see [4] for review). Angiomotin appears to be involved in VEGF signaling in vitro and angiomotin deletion is associated with variable degrees of vascular malformation in vivo [14] although AST seems to have no effect in the same system [15].

There is rapidly expanding evidence that immune system components, in particular the innate immune system, play a key role in induction of angiogenesis in cancer as well as other pathological and physiological conditions (see [16-18] for review), and that innate immune cells are targets for angiogenesis inhibition. We had previously observed that AST inhibited migration of neutrophils and monocytes in vitro and blocked neutrophil mediated angiogenesis in vivo [12]. AST also blocked angiogenesis induced by HIV-tat [19], a molecule with chemokine-like and VEGF-like properties [20]. Angiostatin therapy has been found to reduce macrophage numbers in atherosclerotic plaques [21]. AST inhibits neutrophil and monomyeloid cell adhesion [22], tumor-associated macrophage infiltration in vivo [23], and it inhibits the activity of osteoclasts [24]. While the mechanisms of interaction of AST with innate immune cells are not fully elucidated, recent studies show that AST interacts with CD11b, a component of the Mac-1 integrin [22,25] that is present on neutrophils, macrophages and myeloid derived suppressor cells, in a manner distinct from that of plasminogen.

The effects of AST on cellular immune infiltrates could dictate alterations in the cytokine profile at the local microenvironment or systemic levels following AST treatment. IL-12 is a principal Th1 cytokine that harbors potent anti-angiogenic activity produced by neutrophils, macrophages and dendritic cells. Since AST targets leukocytes that are primary sources of IL-12, we examined the role of IL-12 in AST induced angiogenesis inhibition in vivo. Here we show that the ability of AST to inhibit angiogenesis is dependent on the presence of an intact IL-12 signaling system using multiple knock-out animal models in vivo and that AST induces IL-12 mRNA synthesis in human macrophages in vitro. These data are the first indication of an innate immunity cell product as mediator of angiostatin effects indicating its role in immune cell stimulation rather than direct anti-vascular activity in its antiangiogenic properties. These suggest that a different trial design using angiostatin in cancer therapy or prevention should take into account inflammatory angiogenesis [16].

## Materials and methods

### Angiostatin

Angiostatin used was either purified from human plasma or a recombinant angiostatin produced in *P.Pastoris*, both from Calbiochem. Testing for endotoxin using the highly sensitive Limulus assay indicated only trace reactivity for the purified human material and none for the recombinant peptide.

### Matrigel angiogenesis assay

The assay was performed as previously described [12,26]. Angiostatin or peptides were added to the matrigel sponges at 2.5 μg/ml [12]. In some cases polyclonal antibodies against murine IL-12 (Peproteck, Inc. London) or anti-Phage mouse polyclonal irrelevant antibody (5 prime, 3 prime Inc., Boulder, Colorado) were added at 150 ng/ml. After 4 days the gels were recovered, weighed and processed for hemoglobin quantification or histology as previously described [12,26]. The animals used were either C57bl/6 (Charles River, MI), IL-12Rβ2 KO mice (Jackson labs, the kind gift of Dr. Irma Airoldi, Gaslini Inst, Genova) or IL-12 p40 gene targeted mice (strain B6.129S1-*Il12b*^*tm1jm*^/J; Jackson Labs) on C57bl/6 backgrounds with wild-type littermate controls. KSCM was obtained by incubating sub-confluent cells in serum-free DMEM for 24 hours followed by centrifugation and storage at -20°C. The VEGF/TNFα angiogenic cocktail contained 100 ng/ml VEGF and 2 ng/ml TNFα and heparin (24–26 U/ml). IL-8 (CXCL8) and CCL2 (MCP1) were used at 50 ng/ml. In some cases an IL-12 expression plasmid, or the respective control plasmid, were used in a naked DNA approach where the plasmids were injected into the muscle of mice 2 days prior and on the same day as injection of the matrigel as previously described [26]. Hemoglobin content was measured with a Drabkin reagent kit 525 (Sigma). The data shown were pooled from multiple experiments and normalized to relative controls. For histological analyses, the matrigel pellets were fixed in 4% paraformaldehyde and embedded in paraffin; four μm sections were stained with hematoxylin-eosin by standard procedures.

### Detection of IL-12 following AST treatment in vivo

Thirteen CD1 nude mice were injected with KS-Imm cells and subdivided into 6 mice inoculated peri-tumorally with AST once a week for four weeks at 2.5 μg in a 100 μL volume, and 7 vehicle-treated controls. At four weeks the levels of IL-12 in the sera were analyzed by an ELISA kit (from R&D Systems, Minneapolis, Minnesota).

### Statistical analyses

Statistical differences between individual groups were determined using an unpaired two way t-test (Mann-Whitney) where P values ≤ 0.05 were considered statistically significant. Tumor growth curves were analyzed by two-way ANOVA using Bonferroni posttests to determine significant differences on individual days. Again, P values ≤ 0.05 were considered statistically significant. All data were analyzed using the Prism (Graph Pad) statistics and graphing program.

### Activity of AST on macrophages in vitro

Monocytes were isolated from human peripheral blood using standard Ficoll and Percoll gradients. Cells were put in Petriperm (20 × 10^6 ^in 8 ml RPMI 1640 complete medium with 30% FCS) for differentiation to immature macrophages. After 5 days the macrophages were assessed by morphologic criteria and by FACS analysis with a monoclonal antibody to human CD68. Cells were seeded into two 6 well plates for differentiation. Where indicated, Angiostatin was added 1 hour before a 4 hour treatment with IFNγ (250 U/ml) and LPS (100 ng/ml) to induce differentiation. RNA was subsequently extracted by the TRIzol method (Invitrogen), quantified by optical density (OD) measurement, and checked for quality. c-DNA synthesis was performed from 1 mg c-DNA using T7-(dT)24 and Superscript cDNA synthesis kit (Invitrogen). Real-time PCR reaction was performed using SyBer Green PCR Master Mix (Applied Biosystems) and detected by ABI-Prism 5700 Sequence Detector (Applied Biosystems). Relative expression values with standard errors were obtained using Qgene software and normalized to the expression of the house-keeping gene β-actin. Data were obtained from independent experiments done in triplicate.

## Results

**Figure 1:**
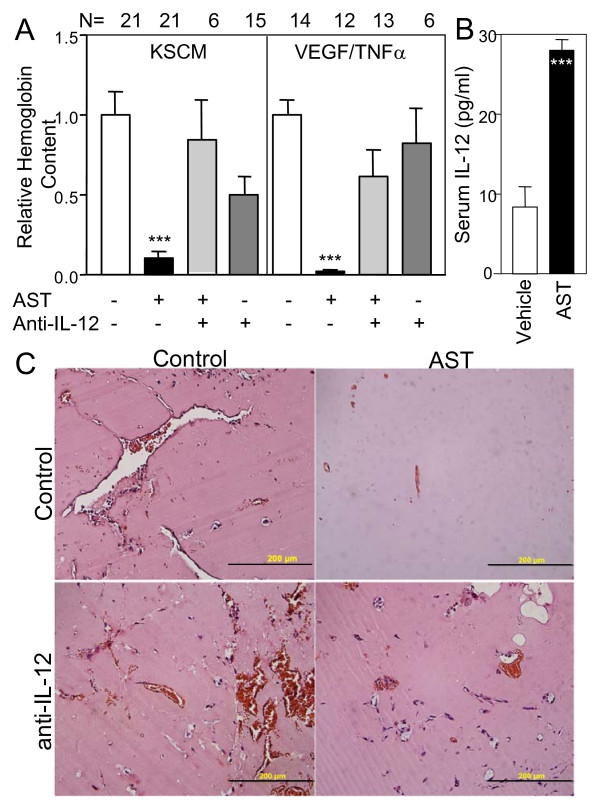
**A: Reversion of angiostatin angiogenesis inhibition by function blocking antibodies to IL-12**. The matrigel angiogenesis assay was performed with the addition of factors as indicated by "+". The angiogenic stimulant was either Kaposi's sarcoma cell conditioned medium (KSCM) or VEGF (100 ng/ml) and TNFα (2 ng/ml) as indicated. AST = addition of angiostatin at 2.5 μg/ml. Anti-IL-12 = addition of 150 ng/ml of anti-IL12 antibodies. Means ± SEM are shown. *** = P < 0.001 (Mann-Whitney) when compared to controls (VEGF/TNFα or KSCM). N = indicates the number of samples in each group. Irrelevant antibodies had little effect on angiogenesis or AST inhibition (data not shown). B: Serum levels of IL-12 found in mice following weekly treatment with angiostatin. *** = P < 0.001 (Mann-Whitney) when compared to control. C: Histology of matrigel sponges. Gels removed at the end of the angiogenesis assay were fixed and paraffin embedded, 4 μM sections were obtained and hematoxylin-eosin stained. Addition of an angiogenic stimulus (KSCM shown) resulted in cellular infiltration and vascularization of the matrigel. The addition of AST strongly inhibited both cellular infiltration and angiogenesis. Antibodies to IL-12 (anti-IL-12) reversed the inhibitory effect of AST on cellular infiltration and vessel formation, but had little effect in control gels. Bar = 200 μm.

Angiostatin (AST) in an angiogenic setting using the matrigel sponge angiogenesis assay in C57bl mice [27] effectively inhibited angiogenesis produced by inclusion of a potent angiogenic cocktails, either supernatants from Kaposi's sarcoma cells or a combination of VEGF and TNFα [26]. The addition of AST at 2.5 μg/ml into the sponges caused a dramatic inhibition of the angiogenesis induced by these stimuli (Fig. 1a, P < 0.001; Mann-Whitney), similar to that observed for AST inhibition of chemokine-induced angiogenesis [12].

### Effects of function blocking antibodies on angiogenesis in vivo

In a preliminary study we noted elevation of serum IL-12 in tumor-bearing animals treated locally with AST (Fig. 1b), suggesting that this potent anti-angiogenic cytokine may play a role in the effects of AST. We therefore tested the effects of function blocking antibodies to IL-12 in vivo. Inclusion of a function-blocking antibody to IL-12 along with AST essentially completely abrogated the capacity of AST to inhibit angiogenesis (Fig. 1a), while the antibody alone had little effect on angiogenesis. Irrelevant antibodies did not substantially affect either the capacity of AST to inhibit angiogenesis or induction of angiogenesis itself (data not shown).

Histological analyses of the matrigel pellets treated with vehicle or AST confirmed the data obtained by hemoglobin quantification. In gels with the addition of AST, few vessels and infiltrating cells were observed (Fig. 1c). In keeping with the results of hemoglobin analyses, the addition of IL-12 blocking antibodies restored cellular infiltration and vessel formation in the gels containing AST (Fig. 1c).

### Role of IL-12 in AST induced angiogenesis inhibition

The IL-12 receptor (IL-12R) is a heterodimer composed of a β1 and a β2 chain, both of which are needed for high-affinity cytokine binding and signal transduction [28,29]. IL-12Rβ1 also forms a heterodimer with IL-23R that acts as a receptor for IL-23, thus only the IL-12Rβ2 subunit is unique to the IL-12 system. By analogy, IL-12 is a heterodimer formed by the p35 and p40 subunits; while the related IL-23 is formed by the IL-12p40 subunit and p19, thus IL-12p35 is unique to the IL-12 signal system while p40 is common to IL-12 and IL-23.

**Figure 2:**
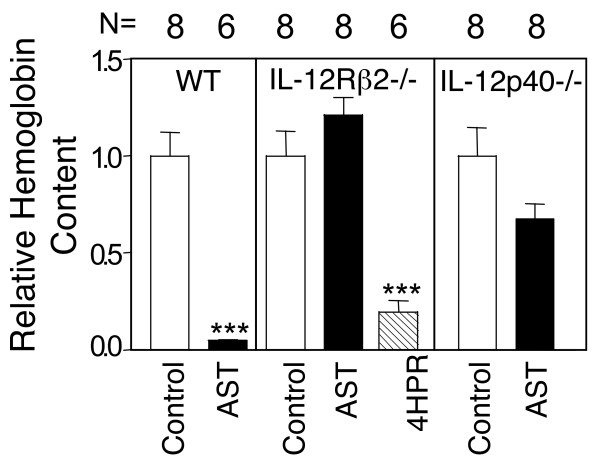
**AST lacks anti-angiogenic activity in animals gene targeted for the IL-12 receptor or for IL-12**. AST was able to inhibit angiogenesis in wild-type (WT) animals but not in animals gene targeted for either the IL-12 specific receptor IL-12Rβ2 (IL-12Rβ2-/-) for the IL-12 signal system or for the IL-12 p40 subunit (IL-12p40-/-). Another angioegensis inhibitor, fenretinide (4HPR), retained anti-angiogenic activity. N = indicates the number of samples in each group. *** = P < 0.001 (Mann-Whitney) as compared to respective controls.

We confirmed the role of IL-12 in AST inhibition using two different murine gene targeted animals. In agreement with the observations using function-blocking antibodies, angiostatin completely lost its capacity to inhibit angiogenesis in IL-12Rβ2 gene targeted animals (Fig. 2). This was not due to inherent defects in angiogenesis inhibition, as Fenretinide (4HPR), an angiogenesis inhibitor with a different mechanism of action [30,31] retained full angiogenesis inhibition activity (Fig. 2). The IL-12Rβ2 gene targeted animals have elevated IL-12 levels that could potentially mask eventual non-IL-12R mediated anti-angiogenic effects. We therefore tested the ability of AST to inhibit angiogenesis in animals gene targeted for the IL-12 p40 subunit. Again, AST completely lost its capacity to inhibit angiogenesis in animals lacking the capacity to produce IL-12 (Fig 2). Taken together, these data demonstrate that IL-12 production and signaling is an integral part of AST angiogenesis inhibition.

### AST inhibits angiogenesis induced by IL-8 (CXCL8) but not by CCL2 (MCP1)

**Figure 3:**
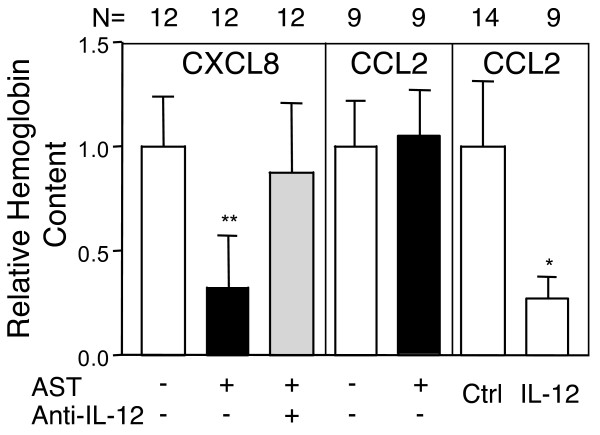
**AST inhibits angiogenesis induced by the chemokine IL-8 but not by CCL2**. AST effectively inhibited angiogenesis induced by IL-8, and this inhibition was reversed by anti-IL12 antibodies. In contrast, AST was unable to inhibit angiogenesis induced by CCL2, while a systemic naked DNA IL-12 approach resulted in effective angiogenesis inhibition. These data indicate that CCL2, which preferentially targets monocytes and macrophages, skews these cells toward a AST resistant phenotype. N = indicates the number of samples in each group. * = P < 0.05; ** = P < 0.01; (Mann-Whitney) as compared to respective controls.

We had previously shown that angiostatin is able to inhibit angiogenesis induced by diverse CXCR2 ligands in vivo in a neutrophil dependent manner [12]. In keeping with these data, angiostatin inhibited angiogenesis induced by CXCL8 (Fig. 3). However, angiostatin did not inhibit angiogenesis induced by CCL2 (MCP1), a chemokine principally active on monocytes and macrophages, while a systemic naked DNA gene therapy protocol using an IL-12 expression vector as previously described [26] effectively inhibited angiogenesis induced by CCL2 (Fig. 3). This suggested that exposure to CCL2 modulates the response of cells targeted by this chemokine, including macrophages and dendritic cells, to AST.

### AST induces IL-12 mRNA expression in macrophages

**Figure 4:**
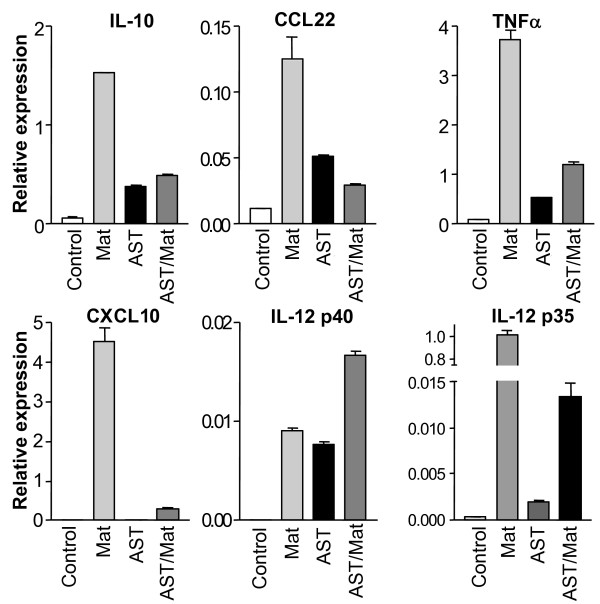
**AST induction of IL-12 mRNA production by macrophages**. Immature macrophages were differentiated from human monocytes in culture and untreated (control) or treated with either AST, a combination of IFNγ and LPS (Mat; Mature), or both as indicated. Real-time PCR analyses of mRNA indicated that AST treatment rapidly induced production of several cytokines including both the subunits of IL-12, similar to that observed after maturation with IFNγ and LPS (with the exception of CXCL10). Treatment with both AST and IFNγ/LPS was additive only in the case of the IL-12 p40 subunit.

We examined the effects of AST on expression of diverse markers for the differentiation of human monocyte-derived macrophages. Real-time PCR demonstrated a 6 hour exposure of ''naïve'' macrophages to AST significantly (P < 0.001 for both, Students t-test) induced expression of IL-12 mRNAs for both the p40 and p35 IL-12 subunits (Fig. 4), in the case of p40 to levels close to that induced by differentiation with IFNγ and LPS. The expression of other markers of differentiated macrophages was also induced by AST alone. Induction of expression of these differentiation makers by a single stimulus to levels at times reaching that of the potent combination of IFNγ and LPS was quite remarkable. Interestingly, the combination of AST and IFNγ/LPS was additive only in the case of the p40 Il-12 subunit (Fig. 4).

## Discussion

Anti-angiogeneic therapy is being increasingly applied in the clinic with important benefits for cancer patients. However, current strategies are principally targeting the key endothelial factor VEGF, which has encountered problems with both tumor escape as well as adverse cardiovascular effects [32]. Immune cells appear to be key mediators of tumor escape mechanisms [33], and thus represent important clinical targets. AST was the first of several endogenous inhibitors of angiogenesis that are fragments of proteins with unrelated activity [1]. While intense research efforts have identified potential receptors on endothelial cells, AST also has clear activity on diverse innate immune cells. Here we demonstrate that induction of IL-12 production is a key component of the anti-angiogenic properties of angiostatin in vivo. Removal of the IL-12 signal cascade by removal of either the ability to produce IL-12 or to respond to IL-12 completely abrogated the ability of AST to inhibit angiogenesis. Further, we show that "naïve" macrophages induce synthesis of the mRNAs for the IL-12 subunits, as well as other cytokines, when treated with AST. Interestingly, CCL2 appears to desensitize mononuclear cells to the effects of AST in vivo, potentially explaining some of the variation in efficacy of angiostatin in observed with different model systems.

We note that many peptide angiogenesis inhibitors identified through functional assays are peptide fragments of proteins that normally have independent functions. The immune system is capable of sensing at least some forms of proteolytically generated peptides [34], a role for the immune system in the function of this class of angiogenesis inhibitors could be speculated, in keeping with the immunomodulatory properties of the calreticulin fragment vasostatin [35]. Thus the role of the immune system as a primary target for endogenous angiogenesis inhibitors may be a broader class paradigm.

CD11b positive infiltrates have been found to be responsible for the resistance of tumors to anti-VEGF therapy [33], largely via production of the angiogenic VEGF-related factor Bv8 [36]. Angiostatin clearly influences the angiogenic potential of neutrophils and macrophages, potentially through modulation of the CD11b/CD18 Mac1 integrin activity [25]. In addition to up-regulation of the anti-angiogenic factor IL-12, it may also repress production of Bv8 and provide a mechanism for blocking tumor escape from anti-VEGF therapies.

## Conclusion

Taken together, our data indicate that when analyzing the activity of angiogenesis inhibitors and searching for clinical anti-angiogenesis targets, the role of bone marrow derived components, in particular the innate immune system, are critical determinates that must be taken into consideration and represent key therapeutic targets.

## Competing interests

The authors declare that they have no competing interests.

## Authors' contributions

AV, GL and MM carried out the in vivo studies. MP, GL and AM carried out the in vitro immunoassays and RT-PCR analyses. AS participated in the design of the in vitro studies. AA, CB, DMN conceived the study, and participated in its design and coordination and drafted the manuscript. All authors read and approved the final manuscript.
